# Agroecological transition or *agroecological repossession*? Resurgence of traditional food culture in the Tremembé da Barra do Mundaú

**DOI:** 10.1177/11771801251369560

**Published:** 2025-09-01

**Authors:** Lauriane Tremembé, Mateus Tremembé, Evan Bowness

**Affiliations:** 1 Indigenous Territory of the Tremembé da Barra do Mundaú, Brazil; 2 The University for the International Integration of Afro-Brazilian Lusophony, Brazil; 3 Centro de Estudos do Trabalho e de Assessoria ao Trabalhador e à Trabalhadora (CETRA), Brazil; 4 Western University, Canada; 5 Associação para Desenvolvimento Local Co-produzido (ADELCO), Brazil

**Keywords:** agroecological transitions, agroecology, environmental repossession, food sovereignty, Tremembé food systems, Western and Indigenous knowledge systems

## Abstract

Industrial food systems, as extensions of colonial dispossession, are destroying the planet. Agroecology is increasingly promoted as a response, framed as an ecological farming practice, a grassroots movement, and an academic field. Yet, much agroecological knowledge remains embedded in the same Western institutions and epistemologies that underpin industrial agriculture. As such, agroecological science can reproduce patterns of knowledge extraction and exploitation. Drawing on *environmental repossession*, which describes how Indigenous communities restore relationships with land, and a framework for ethical engagement between Indigenous and Western sciences, we reframe agroecological transitions as agroecological repossessions. This reframing centers the resurgence of traditional foodways and resituates innovations in ecological agriculture within the continuity of ancestral land relations. Through an example from the Tremembé da Barra do Mundaú (an Indigenous community in Ceará, Brazil), we show how reframing agroecological transitions this way opens the possibility of reclaiming agroecological science itself.

## Introduction: toward agroecological transitions?

Many people think agroecology doesn’t exist, that it’s a utopia, a lie. But agroecology, from the Tremembé [an Indigenous community in Ceará, Brazil] perspective, is possible. From our perspective, from the perspectives of various Indigenous territories, we have been practicing agroecology all along.

—L. Tremembé, Field notes, July, 2024

Industrial food systems—characterized by large-scale, chemically intensive, and highly concentrated agricultural commodity production—are widely recognized as major contributors to climate change, biodiversity loss, pollution, and resource depletion (Crippa et al., 2021; Springmann et al., 2018; Yang et al., 2024). Food systems scholars have also long argued that the mass production of “food from nowhere” (Campbell, 2009, p. 309) cannot continue indefinitely, motivating an increasingly urgent need to *transition* the food system to a more sustainable and equitable means of producing food. More specifically, there is growing recognition that to navigate the complex problems in the food system resulting from commodification, industrialization, financialization, and increasingly, techno-solutionism, societies must transition to food systems that are grounded in the very principles that sustained healthy ecosystems prior to the emergence of the colonial and corporate global food regime (Bowness et al., 2025; Ruder et al., 2022). One alternative gaining traction in international policy discussions is *agroecology* (Food and Agriculture Organization of the United Nations, 2018; International Forum for Agroecology, 2015; James & Bowness, 2021). For our purposes, we define agroecology as a practice, a movement, and a science (Wezel et al., 2009). As a practice, agroecology refers to techniques and farm management decisions applied throughout agricultural landscapes and usually encompasses different skills and methods aimed at enhancing agrobiodiversity, improving soil health, and better managing water. As a social movement, agroecology consists of various organizations, community leaders, and policy actors who mobilize to challenge industrial agriculture by creating initiatives and advocating policies that support farmers and consumers to build more agroecological food systems. Agroecology is also a science with foundations in agroecosystem research, which analyzes the interactions between crops, livestock, soils, and the broader environment to create sustainable systems. Thus, *agroecological transitions*, an important concept in global policy conversations, imply a shift toward a new type of food and knowledge system, despite the fact that by virtue of prioritizing farming practices that are in balance with nature, these transitions are criticized as romanticizing the drudgery of pre-industrial peasant agriculture (Bernstein, 2017).

In addition to championing sustainable farming practices, agroecological movements and scholarship today advance beyond the ecological aspects of food systems into socio-political dimensions, and they pay particular attention to inequities along the lines of social difference (Bowness et al., 2024; Giraldo & Rosset, 2022). In the literature, agroecology is often described as emerging through a horizontal and transdisciplinary construction process known as the diálogo de saberes (participatory dialogue of knowledges between different social groups and ways of knowing) (Martínez-Torres & Rosset, 2014). While this emergent and ongoing dialogue creates space to accommodate alternative, including non-Western, epistemologies, to actually deliver on goals for social justice related to food, proponents of agroecology must still navigate tensions and disparities in knowledge systems (Anderson et al., 2019), primarily because in settler colonial contexts, settler knowledge is prioritized at the expense of Indigenous and other ways of knowing.

Our author team is led by Lauriane Tremembé and Mateus Tremembé, Indigenous community organizers and agronomists from the Tremembé da Barra do Mundaú, an Indigenous community in Ceará, Brazil, who are deeply engaged in preserving and revitalizing their community’s traditional food culture. Evan Bowness is a non-Indigenous racialized settler and Canadian early career academic whose research focuses on agroecology and climate justice. Since 2022, Bowness has been building a collaborative research relationship with the Tremembé da Barra do Mundaú. Our work together is community-directed, centers the struggles and priorities of the Tremembé communities, and is guided by principles of relational accountability, reciprocity, and a commitment to recognizing knowledge as a collectively held resource. This article is the fruit of dozens of conversations, both virtual and in person, most of which took place on the lands of the Tremembé da Barra do Mundaú. Each author has contributed text, some of which took the form of recorded and transcribed conversations, and we all reviewed and revised a Portuguese version before Bowness translated the final version into English. To ensure that the article’s contributions are returned to the community, a Portuguese version has been prepared for publication.

We generally agree with critical food systems scholars that agroecological knowledge is not and should not be confined to Western science and academic institutions. A more generous conception of agroecological knowledge recognizes that insights into sustainable farming practices emerge from the lived experiences of small-scale farmers, Indigenous peoples, and other communities who live in close relation with the lands and waters that sustain them. In other words, the body of academic agroecological knowledge exists largely because of these kinds of community-based knowledge systems. For example, farmers in small-scale and traditional agricultural settings often possess an intimate understanding of their local ecosystems, acquired through generations of experimentation, adaptation, and observation. By *traditional* we mean the knowledge, practices, or ways of life that have been passed down through generations, often within small-scale, community-based settings, with povos e comunidades tradicionais (traditional communities, including Indigenous peoples and other land-based communities, who sustain distinct ways of life rooted in long-standing relationships with the lands and waters of specific territories) being recognized in Brazilian law and policy as groups that maintain distinctive cultural, social, and economic practices tied to specific lands and territories. The intersection of formal scientific research, traditional or local knowledges, and knowledges from social movements is increasingly recognized through participatory research and community-based transdisciplinary collaborations. These approaches, integral to agroecology, involve farmers, researchers, and local communities working together to co-create knowledge. As Gliessman (2018) writes,

New knowledge is constantly being created in our food systems. Farmers are constantly observing and testing new practices, seeds, tools, and relationships. Agroecology researchers are constantly testing and experimenting in order to understand the ecological foundations of food system function and management. Social movements are constantly listening and integrating the knowledge, needs, and points of view of the people at the frontlines of the struggle for food system equity, fairness, and food justice. When these three forms of knowledge are linked (something academics call *transdisciplinarity*), new knowledge is created. And change occurs. (p. 1; emphasis in original work)

However, while such sound bites are laudable, as authors advocating for agroecology, we aim to push beyond platitudes to confront core inequities in the academic sphere. Much of what is recognized as agroecological knowledge is produced through formal research, living in academic journals, whereby the majority of this work is conducted and brought to light by researchers in the Global North using Western methods for Western audiences. Granted, scientific research conducted within academic institutions does play a key role in advancing global understandings of agroecosystems, from soil health and crop diversification to pest management and climate resilience. However, as Gómez et al. (2013) note, approaches that leave out other knowledge systems might also follow colonial patterns “where industrialized countries lead publishing, conduct research studies both in industrialized and non-industrialized countries, and do not publish in non-industrialized countries” (p. 355) and where local research remains highly local not by choice but by access.

It is well-established that the history of Western research across the globe has been extractivist and exploitative of historically marginalized communities and Indigenous peoples and their ways of knowing and being (Smith, 2021). At the same time, we believe it owes a profound debt to those ways of knowing and being, which include the ancestral practices, spiritual beliefs, and cultural traditions of Indigenous peoples and local communities. However, little sustained effort has been directed toward co-construction in the academic sphere (Utter et al., 2021), and traditional knowledges are otherwise often marginalized in other fields of scientific discourse (Stein et al., 2024). Some efforts have been made toward inclusion, but in 2025, we are seeing how even those terms are being targeted largely by non-scientists and for political reasons that will reverse any attention to these issues and the gains made. Therefore, beyond agroecology as a diálogo de saberes, we argue that while an era of *agroecological transitions* is substantially grounded in traditional practices, the term can be enriched through the language of resurgence and repair. Using a case study emerging from a collective and community-directed research project examining an agroecological transition—as a shift away from industrialized food systems toward agroecological food systems—on the Indigenous lands of the Tremembé da Barra do Mundaú, we discuss the complexities at the interface of Western and non-Western epistemologies. We reframe agroecological transition as *agroecological repossession*, a semantic and ideological act that lays claim to both physical space in the territories in which agroecology exists and in academic and policy discourse. First, we see agroecological repossession as a resurgence of traditional ecological food cultures and second as infused with contemporary ecological farming practices. The Tremembé da Barra do Mundaú demonstrate that agroecology is a lived reality that is rooted in the reclamation of ancestral practices and traditional food cultures, and resistance to colonial and industrial land relations. After describing the Tremembé da Barra do Mundaú and agroecological transition underway in the territory, we consider it through the lens of environmental repossession (Richmond et al., 2024) to show how agroecological transitions do not only refer to a shift toward a new food system but are often fundamentally based in the reclamation of traditional food cultures. All descriptions of the Tremembé da Barra do Mundaú are written by the Tremembé coauthors. We conclude with adaptations of Stein et al.’s (2024) cartography of engagements between Indigenous and Western sciences to *gesture toward* what it might look like to repossess Western agroecological science.

## Tremembé da Barra do Mundaú resistance

The Tremembé Indigenous people inhabit three municipalities in the northeastern Brazilian state of Ceará: Acaraú, Itarema, and Itapipoca. The Tremembé da Barra do Mundaú Indigenous Land is situated in Itapipoca, on the west coast, encompassing the four villages of São José, Munguba, Buriti do Meio, and Buriti de Baixo. According to the Brazilian Institute of Geography and Statistics (Instituto Brasileiro de Geografia e Estatística, 2022), approximately 700 Indigenous people reside in this territory, sustaining themselves primarily through artisanal fishing and family farming, practices rooted in their ancestral heritage. For decades, the Tremembé community endured systemic silencing and exploitation by non-Indigenous landowners and squatters, undeniably colonizers, who imposed near-slavery conditions, forbidding the community from building homes or cultivating their land without paying rent. Even when fees were paid, settlers seized half of the harvest as *taxes*, preventing the community from sustaining itself. Discussions of Tremembé ethnicity or land rights were met with threats of violence, further stifling their identity and autonomy. However, in the late 1990s and early 2000s, the Tremembé began reclaiming their ancestral land and identity through collective resistance and cultural revitalization. Their efforts have yielded significant victories, including the establishment of their own Indigenous educational curriculum in 2005, initiation of the process of land demarcation in 2009, construction of an Indigenous school in the same year, access to Indigenous health care in 2012, and physical demarcation of their land in 2018. In April 2023, their territory was formally homologated as Indigenous land by the Brazilian state, and at the time of writing, the federal government was finalizing the process to compensate non-Indigenous residents to vacate the territory.

One of the most notable triumphs across this history was the community’s territorial repossession against *Nova Atlântida* (New Atlantis, a proposed tourist mega-project), once heralded as the world’s largest tourism initiative. With a budget of USD 15 billion, the Spanish-led enterprise planned to transform nearly the entire 3,580-hectare territory into a sprawling tourism complex. The proposed development included luxury hotels, resorts, condominiums, golf courses, and facilities accommodating 100,000 international visitors. Initial construction restricted access to vital resources, such as water and fishing areas, by erecting fences and employing armed guards. These actions led to violent clashes, including arson and the destruction of homes. Despite facing internal divisions, as some community members were co-opted by the developers, the Tremembé reclaimed their territory by dismantling barriers and resisting encroachment. The threats to Tremembé land extend beyond Nova Atlântida and persist today. Real estate speculation, unregulated tourism, and the development of wind farms, including offshore projects, pose ongoing challenges. Nearby tourism activities disrupt fishing practices, desecrate cultural sites, and harm local ecosystems. While no wind farms currently operate within their territory, the nearby Mundaú wind farm, established in 2014, has already degraded fishing conditions and river health, disrupting fish reproduction and causing siltation. Preliminary plans for offshore wind farms along Ceará’s coast further endanger the region.

Despite these pressures, the Tremembé continue to defend their land, culture, and sacred sites. The Tremembé worldview continues to be deeply rooted in intergenerational relationships. Elders, referred to as troncos velhos (old trunks), are celebrated for their wisdom, which guides the community’s actions and sustains their identity. Tremembé youth, while organizing their own initiatives, ground their efforts in the teachings of the elders. During the demarcation process, two women leaders were selected by the elders to represent the community’s collective struggle, emphasizing the prominent role of women in Tremembé society. Women lead in education, family management, rituals, and broader territorial governance. The Tremembé de Itapipoca Indigenous Council (CITI), founded in 2012, coordinates community actions across the villages, reinforcing cultural practices, farming, artisanal fishing, and traditional festivals. CITI also provides avenues for youth to contribute as teachers, university students, and leaders in various projects. Central among them have been efforts to strengthen the community’s food systems, which have proven to be a key strategy for resurgence and reclamation of the Tremembé connection with the land.

### A resurgence of Tremembé traditional food culture

The so-called “transition to agroecology” in my community wasn’t about adopting new practices; it was about naming and strengthening what we’ve always done. Through partnerships with organizations like CETRA [Center for Labor Studies and Worker Support; a non-governmental organization (NGO)] and initiatives like the Florestação [a community project promoting afforestation] project, we found terms to describe our ancestral practices and techniques to protect them from modern pressures . . . . These projects brought valuable tools and knowledge, but they never replaced what we already knew. They enhanced it. (L. Tremembé, Field notes, July, 2024)

All the food and land experiences and practices carried out by the Tremembé people of Barra do Mundaú are recognizable through what we understand as agroecology. To be clear, what could be referred to as an *agroecological transition* pertains to the academic classification of these practices as *agroecological.* However, from the Tremembé perspective, their food and land-based practices are first and foremost what they refer to as “Tremembé Agroecology”, or traditional practices going back generations. Many of these practices can also be viewed as reconstituted forms of what Richmond terms “environmental repossession” (Richmond et al., 2024, p. viii). Chantelle Richmond and colleagues use *environmental repossession* to describe the dynamic, ongoing processes by which Indigenous peoples reclaim and renew their relationships to the land and all its living beings, in spite of colonial dispossession. Four components make up the praxis of environmental repossession: direct action such as occupations, blockades, resistance camps, and boycotts; the rehabilitation of everyday practices including traditional food gatherings; alliance building including collaboration with Indigenous and non-Indigenous partners; and cultural production through art, performances, and narratives. Each of these practices features in the Tremembé struggle over their lands and waters and in the resurgence of their traditional food systems.

For instance, settler farmers in the region have traditionally adopted the habit of clearing and burning to create fields, often deforesting large areas, including mangroves, leading to the disappearance or reduction of several species of fauna and flora. This in turn damages other relationships, including relationships with Tremembé ancestors or the encantados (enchanted beings linked to nature that animate landscapes and sacred places). In 2014, the Tremembé community began participating in the Florestação project, with support from CETRA, in which Tremembé farmers were trained as *agroecology multipliers.* This training allowed them to implement and improve various production techniques, focusing particularly on their productive backyards and fixed areas for family use. This partnership with a settler-based non-governmental organization (NGO), CETRA, which endorses agroecology, facilitated a dialogue with all farmers, encouraging them to cease burning to clear land, taking into account concerns about biodiversity, and promoting alternatives to maintain the productivity of already cultivated soils. These renewed perspectives, combined with the diverse knowledge and ways of producing food, have been maintained by the Tremembé people despite various forms of violence and displacement. Tremembé traditions have been preserved through experiences and oral stories, whether in the collective circles of cassava scraping, bean threshing, or storytelling. As a result of this experience, the Tremembé people have come to understand that their way of living and working can also be understood as agroecology, leading them to value their knowledge more and to be concerned about protecting and maintaining their agro-socio-biodiversity.

### Alliances with agroecology and food as sacred

Collectively, the Tremembé people decided to abandon conventional agricultural methods and engage in a process of resurgence and modification of certain practices in dialogue with agronomists and agroecological organizations, which emphasized and strengthened their traditional methods of preparation, cultivation, and harvesting. This shift generated conflicts, including attempted murders, threats, and persecution of community leaders, as well as tensions with the National Foundation of Indigenous Peoples, which oversees Indigenous land administration in Brazil, as the community began reporting cases of burning and deforestation as environmental crimes. Despite these challenges, the Tremembé people continue to produce food in family- and collective-use areas, using both ancestral practices—such as heritage seeds and traditional agricultural methods rooted in family-based farming—and newly acquired agroecological practices—some learned through exchanges, others in dialogue with formal agronomy university programs. This combination strengthens their sovereignty, autonomy, and food culture, and the Tremembé community celebrates and shares its food, which is clean, fair, and, above all, sacred.

The community also practices sustainable agriculture by focusing on soil management, water conservation, and natural pest control, all while minimizing environmental impact. In soil management, families engage in minimal tilling, plowing just once a year through a partnership with the municipality of Itapipoca. Manual tools such as hoes, sickles, and wheelbarrows are employed from soil preparation to harvest. Fertilization comes from local organic materials like manure from cattle, sheep, and birds, as well as decomposing leaves and wood from forested areas rich in organic matter. To protect the soil further, many agroecosystems maintain a layer of mulch, which helps preserve moisture, suppress unwanted plants, and prevent erosion.

Given the semi-arid climate of the region, careful water management is crucial. Despite challenges posed by limited rainfall, the community benefits from preserved streams, marshes, and water holes that ensure water availability for both native and cultivated plants. Most crops are timed to grow during months of increased rainfall, as irrigation systems are not used. For domestic needs, deep wells maintained by the Special Secretariat for Indigenous Health provide water, while cisterns, built through programs such as the *1 Million Cisterns Program* and the Secretariat of Agrarian Development of Ceará, help store it for prolonged use. In addition, natural water holes support various activities, including fishing and supplying flour mills. While natural insecticides are occasionally used in small-scale gardens, chemical poisons, called *agro-toxins*, are avoided, as they can contaminate water resources, especially since Brazil has approved several pesticides and herbicides that are banned in most other places on the planet (Lopes-Ferreira et al., 2022). Manual weeding ensures that cultivated plants receive sufficient nutrients and water by controlling unwanted plant growth. Together, these integrated methods reflect the Tremembé people’s commitment to balancing traditional agricultural practices with environmental stewardship.

### Recognizing rich biodiversity

The Tremembé territory of Barra do Mundaú is home to a wide variety of natural wonders, supporting countless forms of life, from microorganisms to birds, mammals, fish, and humans. This rich biodiversity has maintained a delicate balance for centuries. The territory is bordered on one side by the sea and on the other by the Mundaú River, which runs through the territory and along its southern and eastern edges. An estuary, the river is brackish and feeds a significant stretch of mangroves along its banks and is replenished by streams and freshwater springs which form an essential fishing area for the community. Here, many Tremembé families catch fish and different types of crabs, including the aratu (mangrove tree crab) and the caranguejo (ground-dwelling mangrove crab). The mangroves also provide materials for traditional medicine, housing, boats, and natural dyes. The sea, another vital fishing area, stretches from the shoreline into deeper waters, and beyond the abundance of food it provides, it is central to Tremembé identity and heritage. As a coastal people, the Tremembé have long built their social and cultural relationships around the sea and its symbols. In addition, streams crisscross the territory, serving everyday needs such as bathing, washing clothes, and watering animals and plants. The territory is situated within the Caatinga biome, characterized by drought-resistant plants and shallow soil, with its shrub fruits like the murici (yellow berries whose pulp is eaten fresh or juiced) and batiputá (small, dark-silverish berries which yield an oil used medicinally and in cooking), as well as guabiraba (aromatic fruits that are eaten raw, made into juice, and used in traditional medicine), ubaia (fruit tree with yellow edible fruit), and jatobá (hardwood tree with edible fruit). These plants are a source of many traditional Indigenous medicines used in sacred rites of praise and respect. Miles of sand dunes separate the forest from the ocean, which are said to be home to the encantados. The dunes are separated into two areas, with the lower dunes being home to native grasses and usable for crop production and feeding livestock, especially donkeys which roam freely, and the mobile dunes shifting with the winds.

### Tremembé agroecological diversity

In the Tremembé da Barra do Mundaú territory, nearly all food produced is for the families’ self-provision or local trading within the community, with only a small portion being sold. Food is cultivated by various practices, many of which are documented in the Participatory Inventory of the Food Culture of the Tremembé people (Tremembé & Pieroni, 2022). Before planting, the soil is prepared using organic fertilizer made from cashew tree leaves, manure, and food scraps. In January, corn, beans, and cassava are planted, while at the end of April, during the winter, potatoes and pumpkin are planted. In addition, traditional knowledge dictates that planting should not occur during the first rains of winter, and that planting during the full moon helps increase yield. For cassava production, this results in better starch and flour content. Other foods grown by the community include tree fruits, including seriguela (oval-shaped sweet red fruit), guava, papaya, cashew, blackberry, acerola (cherry-like fruit), cherry, mango, soursop, pitomba (small sweet orange tree fruit - Native to the north and north-east of Brazil), citruses, coconut, melon, sugar cane, urucum (seeds of the annatto tree used as natural food coloring and originally as body paint), chives, coriander, tomato, pepper, sweet pepper, chili pepper, banana, passion fruit, cucumber, melon, basil, lemongrass, and boldo (a tree whose leaves have a strong aromatic flavor and are used as a culinary herb in the preparation of medicinal teas), among others. This diversity also produces several artisanal preserves and o﻿ther derived products, such as coconut-based cocada (a chewy dessert), dried fruit preserves, farinha (cassava flour), cashew nuts, sugar cane syrup, garapa (sugarcane juice), and teas made from medicinal plants. Some important recipes from the Tremembé food culture include tiquara (cold beverage prepared by diluting cassava flour in water and sometimes with sugar or honey added), timbança (uncooked dessert made from crushed cashew nuts mixed with dark rapadura [brown sugar candy], white cassava flour, and cashew juice), aluá de murici (fermented beverage made from murici fruit or pineapple, sugar or rapadura, and cassava flour), café de milho (corn coffee—prepared with corn grains, coconut, and rapadura which are combined, roasted, and then pounded), grolado (crumbly, coarse mixture of wet cassava dough often mixed with grated coconut and served with fish), and cambica (purée made from sweet potato cooked in fresh coconut milk). Other dishes such as tapioca, mungunzá (sweet porridge made from corn kernels cooked with milk, sugar, cinnamon, and often coconut and peanuts), carimã (fermented cassava mass used to make cake), and cassava gum cake are also important in the community’s food culture—most are consumed with fish.

The Tremembé da Barra people cultivate in various spaces within the territory across diverse agroecosystems with distinct crops, practices, and management methods. These practices are passed down through generations, with each adapting to the land’s needs. Family spaces include backyards and enclosures, where food is primarily produced for self-consumption, sharing, and occasionally for sale. Backyards contain the greatest diversity of crops due to years of production, where plants support one another, and their proximity to homes facilitates management. There are a few collective planting areas in the dune valleys near the ocean referred to as baixas (low-lying coastal or riverine areas; literally, low areas), and near the marsh are family or collective-use areas designated for crops such as coconut and cashew trees, sweet potatoes, cassava, corn, and beans. Before the agroecological transition process, many clearings involved cutting and burning plots of forest annually, leading to ecological imbalances. However, most community members have since adapted, abandoning this practice and incorporating other soil management techniques to maintain annual crops in backyards, enclosures, and collective planting areas. Collective production spaces, reclaimed and located in lowland enclosures, are either divided into smaller plots managed by individual families with mutual assistance, or they are worked collectively, with everyone sharing the harvest. In addition to these production spaces, the community has established the Raízes da Terra (Roots of the Earth; a seed house project providing a collective space to store and conserve seeds, supporting agroecological practices and food security), which serves as a hub for storing, exchanging, and multiplying creole seeds. Although seed storage has long been practiced by individual Tremembé families and in community exchanges, storing seeds was often difficult during droughts. This seed house has helped increase the resilience and diversity of crop varieties and preserve the genetic heritage of both cultivated and native seeds from the forest.

### Traditional food festivals

**Figure 1.**
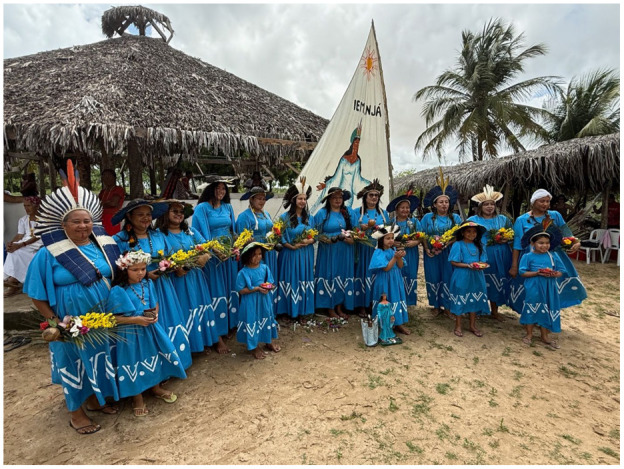
The 2025 Festa de Yemanjá (Photo by Evan Bowness). Festa de Yemanjá = ritual offering gratitude to Yemanjá [the sea goddess] and other enchanted beings of the ocean.

The Tremembé people celebrate their connection to the land through several annual festivals. In January, the community gathers for the Festa de Murici e Batiputá (a festival to celebrate the harvest of the murici and batiputá berries), which began in 2009 to denounce deforestation and fires threatening these native fruits. The festival highlights the importance of murici and batiputá berries for food and income while also showcasing traditional products like murici juice, sweets, and batiputá oil, used in traditional medicine. Just a month later, in February, the community hosts the Festa de Yemanjá (ritual offering gratitude to Yemanjá [the sea goddess] and other enchanted beings of the ocean) for protection of the people and their territory (Figure 1). Held near the river’s mouth, this two-day event includes rituals, offerings, and shared meals as a reaffirmation of the community’s commitment to defending their sacred sea. In July, the community comes together for the Festa da Farinhada (flour-making festival that celebrates the production of cassava flour), a celebration of cassava processing, where families work collectively to produce traditional foods like tapioca, beiju (flatbread or pancake made from glutinous cassava dough baked in a wood-fired oven), and gum cakes, showcasing the cultural importance of cassava throughout the territory. Finally, in October, the Ancestral Food Ritual closes the year of celebrations. Created in 2020 during the COVID-19 pandemic, this ritual honors both the living and the enchanted ancestors by celebrating their legacy through the torém (sacred ritual of song and dance performed in a circle, central to Tremembé cultural identity) and the preparation of ancestral recipes, which symbolize memory, healing, and collective strength. Through these festivals, the Tremembé people not only preserve their traditions but also reinforce their spiritual and cultural ties to the land and their ancestors.

### Cultural groups

Let the elder pass, let the elder pass,

The elder has a hoe, he’s going to work,

He is a professional, he is a worker,

He is an Indigenous man, he is a farmer.

—a song of the sacred Tremembé torém ritual

(Translated by Evan Bowness)

The Tremembé people preserve an intimate relationship with their deities and enchanted beings through rituals and everyday worship. Their main ritual, the torém, involves participants forming a circle to sing, dance, and play instruments, synchronizing their movements and sounds to request protection and honor the enchanted. The Tremembé da Barra do Mundaú is organized around several cultural groups, one of which is the Grupo dos Pequenos Tremembé (Group of the Little Tremembé; cultural group of young children) (GPT), who denounce deforestation and other threats through their torém. The group continually renews its members and is an important means of social and cultural formation for children, who grow up defending and perpetuating their practices. The group sings:

Hey, we are GPT, we want to protect,

Protect nature against the cutting of the mangroves,

Against the lack of respect that the white man shows.

. . .

Hey, we are GPT, we want to protect,

Protect our waters, the springs and the sea,

Against those wind turbines that come to kill the fish.

—a contemporary sacred ritual Torém song, sung by Tremembé children (GPT)

(Translated by Evan Bowness)

**Figure 2.**
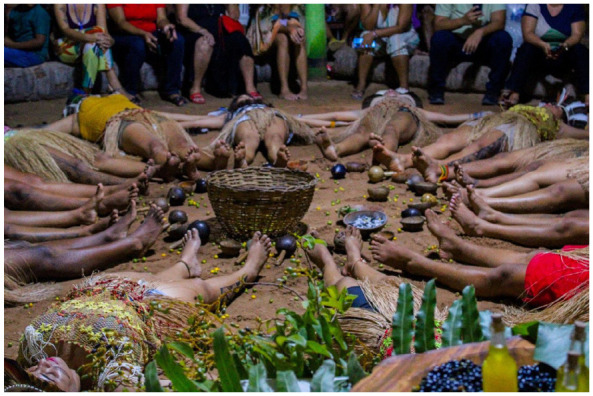
Parente Torém *Ancestral Harvest* performance at Murici and Batiputá Festival 2025 (Photo by Iago Barreto). Parente Torém = youth dance collective founded in 2016 to express the struggle and resistance of the Tremembé [an Indigenous community, Ceará, Brazil] people.

Parente Torém (Youth dance group founded in 2016 to express the struggle and resistance of the Tremembé people) is another cultural group, which represents and makes visible the struggle and culture of the territory through dance, and has several cultural artistic presentations inside and outside the village (Figure 2). In Ekompartir (a performance created by the Parente Torém that explores sharing and cultural expression), one of the youth recites a poem about body painting:

It’s important to believe in what we have in nature,

There’s the mangrove, the jenipapo [fruit of the Genipap tree],

The urucum, such riches,

We collect their liquid,

Transforming art into beauty.

The paintings are all different,

Each one carries meaning,

There’s love, there’s expression,

There’s value and emotion,

There’s the power of spiritual enchantment,

Symbolizing union.

—a contemporary Tremembé poem

(Translated by Evan Bowness)

Another group is Protegidas de Orixás (Protected by the Orishas—divine spirits of Afro-Brazilian religions honored in healing rituals—a women’s collective formed in 2019 to strengthen their struggle through ritual connection with the orixás [orishas], whose performances are spiritual rituals rather than shows). Protegidas de Orixás is a collective of women dedicated to the practice of healing, cleansing, and uplifting rituals, bringing reverence to the enchanted ones and all the forces of nature. In their songs, they bring feminine strength as the greatest link with Mother Earth and various healing prayers and blessings from enchantment. In one of their songs, called *Women’s Empowerment*, the collective narrates the protagonism of Tremembé women:

Woman is an enchanted being, and strength is her legacy,

But if one day she is defied, the world will turn and turn,

For she was not born to be tamed.

I am a daughter of beautiful Jurema [the motherly spirit], protecting my children is my dilemma,

But if one day I am challenged, the world will turn and turn,

For I was not born to be tamed.

—a contemporary sacred ritual song, performed by Tremembé women (Coletivo Protegidas de Orixás)

(Translated by Evan Bowness)

Defensoras da Mãe Terra (Defenders of Mother Earth; a cultural group of Tremembé women who use dance and song to honor Mother Earth) is another group of women who, in dance and music, address the people’s food culture and ways of life, bringing different elements and ancestral foods, and emphasizing the importance of Indigenous women in these processes. In the song *Singing Mother Earth*, they talk about the importance of the Earth and the food that comes from it:

We are seeds of the earth, we sprout from the soil that makes us grow,

We are the roots of the plants that heal and set our air free

Hail to our warriors, from our sacred grounds we are the keepers,

We are fruits of this land, and we want to honor our Mother Earth,

She gives us food, abundance, and sustenance to keep us alive,

We plant and harvest from the land, sacred foods to nourish our lives.

—a contemporary Tremembé women’s song

(Translated by Evan Bowness)

Traditional foods and the lands and waters from which they come hold a vital place in the work of each of these cultural groups, serving as both a source of sustenance and a means of connection to the Tremembé ancestors. Through songs, dances, and rituals, these groups highlight the significance of food not only as nourishment but also as a means of preserving cultural identity, honoring nature, and resisting threats to their way of life.

## Conclusion: toward repossessing Western agroecological science

Historically, academia has extracted knowledge from communities, redefined it, and returned it to us top-down as something new. This perpetuates a hierarchy where our practices are seen as primitive until validated by external frameworks . . . .That’s why it’s vital for those of us in territories to engage with academia on our terms, to speak about what is ours, to strengthen and share our practices authentically. It’s not about commodifying our knowledge but preserving and respecting it . . . . Indigenous practices are rooted in observation, experimentation, and adaptation—just as science is. When we observe the growth of plants, the behavior of animals, the changing seasons, we are conducting research. Yet, our research isn’t valued unless it’s filtered through academic frameworks. This must change. Indigenous people are researchers, scientists, and innovators in our own right, and we deserve recognition as such. (L. Tremembé, Field notes, July, 2024)

The resurgence of traditional food culture and systems in the Tremembé da Barra do Mundaú is more than an agroecological transition, and in this article, we extend the concept to propose *agroecological repossession*. Through our illustration of Tremembé da Barra do Mundaú lifeways, we demonstrate how these communities have reclaimed their ancestral lands and revitalized their ancestral foods through the praxis of environmental repossession. This praxis is varied and includes direct action in opposition to tourism, real estate speculation, and energy development; the shift from slash-and-burn farming, and the return to collective planting areas, native species and heritage seeds, and artisanal fishing and animal husbandry. These practices are all celebrated in rituals, songs, and performances, like the torém, in cultural groups, and during traditional festivals.

These repossessions are also supported by alliance building with settler-based NGOs such as CETRA, government agencies, researchers—including Western scientists, and other Indigenous and non-Indigenous partners. Together, these alliances support the community to iteratively redefine agroecology in ways that align with their priorities. At the same time, agroecology and Western science resonate with Tremembé priorities but are also strengthened through recognition of Tremembé land relationships and ways of being.

As the authors of this article, we each have formal academic training in agroecosystems. However, even within academic institutions, there has emerged a wider recognition of the critical role that Indigenous knowledges play in sustainability (Tom et al., 2019). Alongside this recognition, there is a growing range of ways in which Indigenous knowledges and research intersect (Stein et al., 2024). Increasingly, Western-trained scientists are engaging with Indigenous sciences, and through their work together, they have been addressing the colonial foundations of science while tapping into multiple knowledge systems that help communities and researchers address complex social and ecological crises. Sharon Stein and colleagues offer a social cartography to map these kinds of engagements, and in doing so, they call for a reparative approach to knowledge systems interfaces that emphasizes material and relational repair and Indigenous self-determination. This cartography can be adapted to food systems research (Table 1) to *gesture toward*, as Stein and colleagues (2024) propose, what research collaborations and visions might look like under agroecology repossessed.

**Table 1.** Approaches to food systems research.

	Conventional agri-food systems research	Mainstream agroecology research	Agroecology repossessed
Engagement with Indigenous Food Systems	Indigenous food systems are “primitive” and inefficient	Indigenous food systems contain benefits, but their impact must be verified by Western agricultural science	Food systems research and organizing should redistribute resources to Indigenous communities in support of resurgence
Understanding of Western agricultural and knowledge systems	Western agricultural and knowledge systems maximize productivity, are universally applicable, and are the standard by which all other systems are judged	Western agricultural and knowledge systems are the most advanced but can be improved by incorporating elements of Indigenous food systems	Western agricultural systems are based in one of many knowledge systems, valuable in some contexts but also implicated in ecological degradation and colonial harm
Understanding of the role of agriculture in ecological unsustainability	Western industrial agriculture is the only viable way to feed the world and address ecological challenges	Western industrial agriculture needs to transition to agroecological farming systems to curb environmental impact	Respecting Indigenous sovereignty is the best path to reducing agriculture’s impact on ecosystems
Understanding of the role of agriculture in colonialism	Increasing food production through conventional methods reduces inequality and increases food security	Agricultural systems have been shaped by colonialism, but the current focus is on inclusion and productivity, rather than dwelling on the past	Western agriculture continues to cause the dispossession of Indigenous lands, and reparations are essential for just food systems

Source: Adapted from Stein et al. (2024).

Reflecting on the possibility of repossessing agroecological science, the following concluding thoughts are offered to agroecological researchers and students: Engage with communities on their terms. Understand their struggles and respect their knowledges—and this comes with preparation, so do the work. When you enter a territory to study agroecology or climate adaptation, do not separate practices and ideas from the people, the land, or the spirituality that sustains it. These are all interconnected. And above all, recognize that your role is to support—not to lead—the co-production of agroecological knowledge.
